# Iconography of Beans and Related Legumes Following the Columbian Exchange

**DOI:** 10.3389/fpls.2022.851029

**Published:** 2022-03-14

**Authors:** James R. Myers, Alice K. Formiga, Jules Janick

**Affiliations:** ^1^Department of Horticulture, Oregon State University, Corvallis, OR, United States; ^2^Department of Horticulture and Landscape Architecture, Purdue University, West Lafayette, IN, United States

**Keywords:** Fabaceae, *Vigna*, *Canavalia*, crop dispersal, crop history, plant iconography, *Phaseolus*, common bean

## Abstract

Common bean (*Phaseolus vulgaris* L.), maize, and squash were described by explorers as early as 1492. The illustration of common bean recognized as the first in Europe is in Fuchs’ *Di Historias Stirpium*, published in 1542 and a half-century after beans were observed in the Caribbean. Besides herbals and herbarium specimens, the sources of information on the introduction of New World crops are paintings and illustrations. Two early sources of images of maize and squash are the *Grandes Heures d’Anne de Bretagne* and the Loggia di Amore e Psiche in the Villa Farnesina, Rome. The former was illustrated between 1507 and 1508 and has an image identified as the common bean. The Villa Farnesina Loggia was decorated in 1515–1518, with festoons containing three instances of bean pods. Our first objective was to evaluate these images to determine whether they represented depictions of common bean earlier than the illustration by Fuchs. Neither image appears to be a common bean based on a combination of botanical characters and size. Folio 194 of the *Grandes Heures d’Anne de Bretagne* is most likely a *Vigna* species in the Ceratotropis subgenus. In the Loggia, one set of pods appears to be a species in the Mimosoideae subfamily and the second and third sets of pods most closely resemble *Canavalia gladiata*. Neither image likely represents common beans and are probably Old-World species. Secondly, illustrations of common beans from ten early herbals were analyzed for traits that are characteristic of the centers of domestication and races of common beans. Our objective was to characterize the diversity observed among herbals and determine whether beans from both centers of domestication were present. We potentially identified both Middle American, race Mesoamerica and Andean, race Nueva Granada types. We posit that both Middle American and Andean types were in the Caribbean at the time of the Columbian exchange and that beans from both centers were informally introduced into Europe early on. This review of 16th-century manuscripts and illustrations has provided some answers to the questions of what and when common beans reached Europe and provide new hypotheses for researchers studying the origins, diversity, and distribution of this crop.

## Introduction

Almost immediately upon arrival into the Caribbean in 1492, Christopher Columbus and his men observed the indigenous peoples of the Caribbean growing crops different from what they were accustomed to in southern Europe. While starchy root crops predominated (Sauer, 1952, 1966), the explorers also noticed maize (*Zea mays*), squash (*Cucurbita* spp.), and beans (*Phaseolus* spp.). The 4th of November 1492 entry in Columbus’ navigation journal (Anonymous, 1892) indicated that the inhabitants of Cuba “…tienen faxones y habas muy diversas de las nuestras…” […have cowpeas and broad beans more diverse than ours…]. Columbus was obviously relating the grain legumes he encountered to those with which he was familiar from the Old World. “Faxones” is most likely a reference to common beans because of the similarity in appearance to cowpeas (*Vigna unguiculata*) whereas “habas” was probably the larger, flatter-seeded lima bean that is more similar in size and shape to broad beans (*Vicia faba*) (Hammer, 1992). A second journal entry on November 6 repeats the same phrase. In his final voyage in 1502, Columbus arrived on the Central American coast of present-day Honduras and encountered beans as a staple food crop that were red and white in color as recounted by his son, Ferdinand (Sauer, 1966).

There are many questions about the introduction of New World beans into Europe including when and where were they introduced and where did they come from? What were the characteristics of these beans? What center of domestication did they represent? Some of these questions can be investigated by examining the iconography of the food crops produced after the Columbian exchange began.

The first definite evidence of the introduction of common beans into Europe is found in the mid-16th century. This is roughly 40 years after the Columbian exchange began, so it is not clear exactly when it was introduced or how quickly it diffused through Europe after 1492.

Prior studies of the first images of New World Vegetables in Europe have focused on maize and squash (Janick and Caneva, 2005; Paris et al., 2006). Maize is morphologically very different from Old World cereals, and there is little ambiguity in its identification. Squash presents a greater challenge because cucurbits have been domesticated in both the Old and New Worlds. However, the flowers and fruits show morphological differences that can be used to substantiate the presence of New World pumpkins and squash in Old World iconography (Paris et al., 2006). Large-seeded grain legumes can be challenging to identify in non-botanical works because the differences among genera and species can be quite subtle, especially if only pods are available for analysis. One approach to overcoming this limitation is to employ morphometry for estimating size as well as the subtler characters of pods.

Common bean is one of the five domesticated *Phaseolus* species out of a genus of about 70 species that must be considered in the context of the introduction of New World grain legumes into Europe. The other domesticates are tepary bean (*Phaseolus acutifolius*), runner bean (*P. coccineus*), year or year-long bean (*P. dumosus*), and lima bean (*P. lunatus*). *P. vulgaris*, *P. acutifolius*, *P. coccineus*, and *P. dumosus* (formerly *P. polyanthus*) belong to the Vulgaris group within sister clade B whereas *P. lunatus* in the Lunatus group is more distantly related (Delgado-Salinas et al., 2006). These species became isolated from one another ∼2–4 milliion years before present (MYBP). As indicated by both its vernacular name and Latin-specific epithet (*vulgaris*), the common bean was and is the one most widely distributed throughout North and South America. It is a predominantly autogamous annual adapted to moderate temperatures and precipitation. Of the other domesticated *Phaseolus* species, these have a combination of more specific environmental adaptation and a limited region of production. *P. dumosus* (year or year-long bean) is highly photoperiod sensitive and is rarely seen outside of a narrow zone in the cool, humid tropical highlands of Guatemala. Likewise, the distribution of *P. acutifolius* (tepary bean) is restricted to the hot and dry environments of northern Mexico and the southwest United States. Two species showing wider distributions and more likely to have occurred with common beans in the Latin America-Caribbean region at the time of the Columbian Exchange are lima beans and runner beans. Runner bean is a predominantly allogamous perennial species with tuberous roots that is adapted to the cool and humid tropical highlands (Debouck, 1996; Bitocchi et al., 2017). Lima bean is a predominantly autogamous, annual, and perennial species that has two centers of domestication located in northern South America in the region of present-day Colombia, Venezuela, and Panama, and an Andean center in the area of Peru and Bolivia. It is the second most widely distributed domesticated *Phaseolus* species after common beans. Accessions from the two centers show morphological differences (especially seed size) as well as general adaptation to warm and humid tropics. The two gene pools differ in that the large-seeded Andean “Big Lima” types were traditionally found along the mid- to southern Andes above 1,600 masl whereas the small-seeded types were found along the northern Caribbean coast and valleys of South America below 1,200 masl (Debouck, 1996). The small-seeded gene pool can be further divided based on morphology into “Potato” and “Sieva” types, with the former found in Middle America and the latter distributed throughout the Caribbean in pre-Columbian times (Esquivel et al., 1990).

Wild *P. vulgaris* shows a near contiguous range from Chihuahua, Mexico, through Central America, and extending along the Andes as far south as San Luis, Argentina (Debouck, 1996). Like lima beans, common beans also has two centers of domestication: Middle America with the origin likely being the Lerma-Santiago river valley or Oaxaca region in Mexico and the Andes of central-southern Peru (Kwak and Gepts, 2009; Bitocchi et al., 2017). The domesticates from these two regions are biochemically and morphologically distinct, and furthermore, have been classified into three Andean races (Nueva Granada, Chile, and Peru) and four Middle American races (Mesoamerica, Durango, Jalisco, and Guatemala) (Díaz and Blair, 2006; Blair et al., 2007). Cultivars within races share certain morphological characteristics which can be used to differentiate them from other races (Singh, 1988; Singh et al., 1991).

Additional sources of historical images are illustrations and paintings. These can provide information about the presence of particular crop species at a particular point in time, as well as reveal what morphological characteristics that crop might have had that differs from what is seen today. Non-botanical illustrations may be more difficult to analyze because of artistic license, only a portion of the plant is shown (the fruit may be illustrated, for example, but vegetation and flowers are missing), the material may be arranged for aesthetic rather than documentary purposes, and the objects in an illustration may not be all be of the same scale.

Two images are known that predate the first illustration of common beans in Fuchs’ (1542) herbal, *De Historia Stirpium*, and have been suggested to be the earliest images of common beans in Europe (Camus, 1894; Caneva, 1992). These are in the *Grandes Heures d’Anne de Bretagne* (Book of Hours of Anne of Brittany) (Bourdichon, 1503–1508; de Bonnot, 1979) and the festoons found in the Loggia di Amore e Psiche (Loggia of Cupid and Psyche) in the Villa la Farnesina in Rome (Caneva, 1992). An objective of this manuscript is to independently evaluate the images reported as common beans in the *Grandes Heures d’Anne de Bretagne* and the Villa Farnesina using morphometric methods. Secondly, we examine other illustrations of common beans from the 16th century to understand when and from where its introduction into Europe occurred. Our hypothesis is that the diversity of beans in Europe originated from both common bean centers of domestication from the very beginning of the Columbian exchange.

## Materials and Methods

### Source of Images

The *Grandes Heures d’Anne de Bretagne* is catalogued as Ms. Latin 9474 at the Bibliothèque Nationale de France and can be viewed online in its entirety (Bourdichon, 1503–1508). The images in the *Grandes Heures d’Anne de Bretagne* also illustrate various invertebrates as well as imaginary animals. Many of the insects are identifiable as species common to medieval Europe. In the Folio 194 image, which is purported to be common beans, a beetle in the family Coccinellidae is depicted and was used as a size reference for the plant parts.

Two main sources of images of the Loggia di Amore e Psiche were available for our analyses. These are the Virtual Loggia (Sgamelotti and Caneva, 2017) and Villa Farnesina – Soffitto della Loggia di Amore e Psiche in GigaPan (Di Iorio, 2017). The Villa Farnesina pod images were scaled using adjacent pomegranate, quince, and/or apple fruit. These various fruits are numerous and scattered throughout the festoons with near-uniform size throughout. Based on the sizes determined by Janick and Caneva (2005), we used 10 cm for pomegranate and quince, and 7.6 cm for apple.

In the last decade, a number of old European herbals from the 15th century onward have been digitized and made publicly available. We systematically searched for digitized herbals of the prominent herbalists of the 16th century, using Arber (1953) as a guide for the selection of individuals. Other sources of information on herbals that included bean illustrations included Hedrick (1919), Zeven (1997), Krell and Hammer (2008), and Piergiovanni and Lioi (2010). Herbals were then examined for images of grain legumes in general and *Phaseolus* species in particular. Where herbals were digitized with searchable text, we used search terms that varied depending on the language: Smilax, Dolichos, Phasioli, Phasioln (Latin and Italian), Welch-, Welsch-, or Welsche-Bonen, Phaseolus (German), and Faba or Haba, Frijol, Frijole, and Faxones (Spanish).

### Approach to Evaluating Images

Whereas most other vegetables and fruits are morphologically distinct, many legume pods look superficially similar, and distinguishing the pods of various cultivated large-seeded grain legumes can be problematic. If pictures are sufficiently detailed and enough plant parts are visible, then identification at least to the genus level is possible. Our approach to analyzing the images was to first determine the size of the images, then thoroughly describe the material, looking for key diagnostic features. We subjected pods to analysis using the Intkey Legume Fruits and Seeds electronic-based key (Kirkbride et al., 2003). Genus-specific monographs were also consulted.

Putative images of common bean in herbals were identified and compared for diagnostic morphological traits to verify that the image in question was *Phaseolus* spp., and not *Vigna* or *Dolichos* spp. Unlike the *Grandes Heures d’Anne de Bretagne* or Villa Farnesina images, morphometric scaling was not possible but images were compared for the relative size of plant parts (especially leaf and pod size), colors of flowers and seeds, as well as growth habit and various morphological characteristics. To distinguish *Phaseolus* species from other grain legumes, we looked for the absence or greatly reduced stipules, the presence of stipels on the petiole, absence of a prominent calyx, and presence of bracteoles attached to the base of the corolla. The domesticates from the Middle American and Andean centers of domestication are morphologically distinct, and morphology can help separate the three Andean and four Middle American races. Cultivars within races share certain morphological characteristics which can be used to differentiate them from other races (Singh, 1988; Singh et al., 1991). We classified illustrations from herbals as to race based on the following characteristics: growth habit using the centro internacional de agricultura tropical (CIAT) architectural classification system (Singh, 1981), leaf size, central leaflet shape, bracteole size and shape, beak or stylar spur position, number of flowers or pods per inflorescence, number of seeds per pod, and seed shape (Singh et al., 1991). Not all traits could be used, and in some cases, detail in the image was lacking. For example, some illustrations lacked flowers and others did not display seeds.

The authors of herbals (particularly the later ones) often borrowed illustrations from other authors and these were sometimes mixed with new and original ones. This is particularly the case with Fuchs’ herbals where a number of authors reused his woodcuts (Arber, 1953). We initially classified images without regard to whether they duplicated prior works, then examined for similarities. This provided a check on the accuracy and repeatability of classification for various traits.

## Results

### Folio 194, *Grandes Heures d’Anne de Bretagne*

Anne de Bretagne (1477–1514) Duchess of Brittany and twice queen of France, commissioned the artist, Jean Bourdichon (1457–1521), to create a prayer book for her personal use (Camus, 1894). The *Grandes Heures d’Anne de Bretagne* was illustrated in Tours, France between 1503 and 1508 and shortly after the discovery of the New World. Many pages contain a prayer surrounded by a vertically oriented image of a plant with some animals present. Over 300 species of plants are depicted and include examples of native flora from the Touraine region in northwestern France as well as ornamental and food plants from the Royal Gardens of Tours and Blois (Camus, 1894; Bilimoff, 2001). The animals included in the illustrations range from invertebrates common to the region, to indigenous vertebrates, to fantastical organisms. Paris et al. (2006) analyzed the cucurbit images in the *Grandes Heures d’Anne de Bretagne* and concluded that one is *Cucurbita pepo*, supporting the idea that New World crops were present at the time that the book was illustrated. Paris et al. (2006) provide an in-depth discussion of the prayer book and its various manifestations to which we refer the reader for more detail.

**FIGURE 1:**
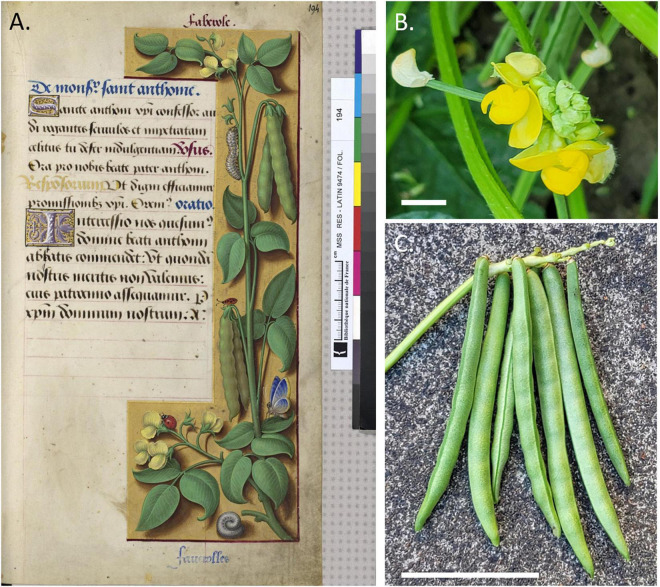
Image from the *Grandes Heures d’Anne de Bretagne* of a putative illustration of common bean and the possible species the image actually represents. **(A)** Digitized image of Folio 194 from Ms. Latin 9474 at the Bibliothèque Nationale de France. **(B)** Adzuki bean (*Vigna angularis*), representative of the subgenus Ceratotropis - flowers (size bar = 1 cm) and **(C)** Raceme with green pods (size bar = 5 cm). Photos: J. R. Myers.

Folio 194 of the *Grandes Heures d’Anne de Bretagne* was described by Camus (1894) as being *P. vulgaris*. The image is in a reverse C shape surrounding the text, with the inscription in Latin “Faberole” above, and the vernacular French “Faverolles” below the image (Figure 1A). The image shows a portion of a leguminous plant with five insects also present. These include one adult and two lepidopteron larvae, a ladybird beetle (family Coccinellidae), and a true bug (possibly family Corizidae). The plant shows a single stem with approximately six nodes, the terminal node being reproductive, characteristic of a determinate growth habit. In some cases, inflorescences arise from a leaf axil, alternately, the inflorescence arises directly from the main stem. The plant has trifoliolate leaves on relatively long petioles and with the terminal leaflet subtended by a rachis. The leaves and stems are glabrous and have a fleshy and perhaps glaucous appearance. The plant lacks stipules and stipels. Leaflets are ovate with acute or acuminate tips and are slightly asymmetrical in appearance. The plant has two pairs of mature pods at two nodes and inflorescences with open flowers at three other nodes. The lower inflorescence apparently arises from a leaf axil, but the three inflorescences on the upper stem arise directly from the stem. The lower inflorescence has six flowers visible, and a broken stem that may be an additional flower. One of the six flowers lacks a corolla with only the calyx visible. Each of the three upper inflorescences has two flowers each. The top inflorescence consists of an unopened flower with only the calyx visible, and a second newly opened flower with the banner petal not fully reflexed. The second from the top inflorescence has two fully opened flowers, and the third inflorescence shows the calyx only; presumably, the spent blossoms have dropped. The flowers are uniformly yellow with large reflexed banner petals, and fairly short and broad wing petals. The keel is not visible in any of the flowers. The corolla is subtended by a calyx consisting of a set of three or four visible sepals arranged with apparent radial symmetry. No bracteoles are visible subtending either flowers or pods. Pods are straight with seed constrictions externally visible and have a short, placentally oriented spur. Pods appear to be green but physiologically mature with seven to nine ovules. Pods are apparently round in cross-section.

The pod and flower dimensions were estimated using the insects in the image to determine the scale. Because it was the least ambiguous in terms of identification and known dimensions, we used the ladybird beetle as the basis for all measurements. Ladybird beetles range in size from 0.8 to 11 mm with most European species in the range of 5–10 mm (Arnet et al., 2002); we chose an intermediate length (head, thorax, and abdomen) of seven millimeters. Based on these dimensions, we determined that the pods range in length from 37 to 44 mm with a width of 4.7 to 7.0 mm (Table 1 and Figure 1A). Flowers are from 7.0 to 11.7 mm in height and 4.7 to 10.5 mm in length.

**TABLE 1:** Pod and flower measurements^1^ from folio 194, *Grandes Heures d’Anne de Bretagne.*

Pod position	Length (mm)	Width (mm)	No. ovules	L:W ratio	
Top front	44.3	7.0	9	6.3	
Top back	40.8	5.8	7^2^	7.0	
Lower front	40.8	7.0	8	5.8	
Lower back	37.3	4.7^2^	7	8.0	

**Upper raceme**			**Lower raceme**		
**Flower position**	**Height (mm)**	**Length (mm)**	**Flower position**	**Height (mm)**	**Length (mm)**

Upper (inverted)	7.0	4.7	1^3^	10.5	10.5
Lower left	11.7	9.3	2	–	7.0
Lower right	7.0	7.0	3	11.7	10.5
			4	–	–
			5	8.2	9.3

*^1^Scaled to ladybird beetle in the image where the length of the body (excluding antennae) is estimated to be 7 mm.*

*^2^Estimate, pod partially obscured.*

*^3^Clockwise beginning with bottom flower.*

Overall, the image depicted in Folio 194 is not completely consistent with any single grain legume species. This is somewhat surprising because other grain legumes depicted in the *Grandes Heures d’Anne de Bretagne* (*Cicer arietinum*, *Vicia faba*, *Pisum sativum*, and *Lathyrus sativus*) are quite accurate and easily recognizable. Perhaps the artist was unable to see the plant in all stages of its lifecycle and borrowed from other legume species to fill in the blanks. The lack of conspicuous stipules on the stems is consistent with *Phaseolus*, but also with some Old World species. A lack of stipels on the leaves would argue against *Phaseolus*, but this detail is often left off in illustrations of this species. The presence of a prominent calyx and the absence of bracteoles also argue against *Phaseolus*. The exaggerated bumpiness over the ovules in the pods is not consistent with any cultivated grain legume. For some traits, there appears to be a blending of characters from different species. The trifoliate leaves and determinate habit are consistent with both *Phaseolus* and *Vigna* spp., but the flowers, pods, and glabrous vegetative appearance more closely resemble that of *Pisum sativum* rather than any warm-season legume. None of the cultivated *Phaseolus* species have yellow flowers; rather, they are white to purple in color. Among grain legumes, only those species belonging to the subgenus Ceratotropis of the genus *Vigna* possess solid yellow flowers. These include cultivated Asian species *Vigna aconitifolia, V. angularis, V. glabrescens, V. mungo, V. radiata*, and *V. umbellata*). The flowers and pods of *V. angularis* (adzuki bean) are shown in Figures 1B,C.

Based on 19 pod characters in InKey, (the specimen belongs to subfamily Faboideae; the fruit is a unilocular legume 4.5 cm long and 0.7 cm wide and thick, and 2–9 times longer than wide; the fruit has a deciduous corolla but a persistent calyx, and the calyx is shorter than the fruit; the fruit is straight without orifice formed by the curving of the fruit; the fruit is not plicate or twisted and is asymmetrical with both sutures nearly straight; the fruit is terete but not inflated and has a spur), the *Grandes Heures d’Anne de Bretagne* specimen keys out to the genus *Vigna* (Kirkbride et al., 2003). Assuming accurate scaling, flower and pod size is more in line with *Vigna* spp. than other grain legumes (Table 2). The one pod character that is not consistent with Vigna is that it has a blunt apex with a short spur; Most *Vigna* species have a long, tapered apex and spur. We conclude that the plant depicted in Folio 194 of the *Grandes Heures d’Anne de Bretagne* is not *P. vulgaris* as initially suggested by Camus (1894). It is most likely a *Vigna* species in the Ceratotropis subgenus. Because of lack of detail and botanical inaccuracies, it is impossible to identify this specimen to the species level.

**TABLE 2:** A comparison of the plant depicted in Folio 194 of the *Grandes Heures d’Anne de Bretagne* with several large-seeded legume genera.

Subject	Growth habit	Insertion of inflores-cence	No. flowers/inflores-cence	Flower length (cm)	Flower color	Calyx	Leaves	Ten drils	Pod length (cm)	Pod width (cm)	Seeds/pod (no.)	Stem and foliage appearance
Folio 194	Determinate	Leaf axil and internode	3–6	0.5–1.1	Yellow	At least 4, probably 5 free sepals	Trifoliolate; ovate w/acute or acuminate apex	Absent	3.7–4.4	0.5–0.7	7–9	Glabrous and glaucous
*Phaseolus vulgaris*	Determinate and indeterminate	Leaf axil	4–12	1.0	White, pink purple	Minute toothed calyx obscured by a pair of bracteoles	Trifoliolate; ovate with acuminate apex	Absent	8.0–20.0	1.0–1.5	4–12	Lightly pubescent
*Vigna* spp. (subgenus Ceratotropis)	Indeterminate only?	Leaf axil	5–12 (10–25 in *V. radiata*)	0.9	Yellow	Toothed calyx larger than *P. v.* but hidden by a pair of bracteoles	Trifoliolate; ovate to ovoid-lanceolate; acute to acuminate apex	Absent	2.5–12.5	0.4–0.6	4–12	Lightly to heavily pubescent
*Vigna unguiculata* (subgenus Vigna)	Determinate and indeterminate	Leaf axil	2–4	2.0–3.0	White, pink, blue, violet, dirty yellow	Toothed calyx larger than *P. v.* but hidden by a pair of bracteoles	Trifoliolate; ovoid-rhomibic; sometimes sl. lobed; acute apex	Absent	10.0–100.0	0.3–0.5	10–20	Lightly pubescent
*Pisum sativum*	Indeterminate only	Leaf axil	1–3	2.0–3.0	White, pink, purple	Five free sepals; no bracteoles present	Pinnate with 1–3 pair of leaflets	Present	4.0–12.0	1.5–2.0	2–10	Glabrous and glaucous

### Images From the Loggia di Amore e Psiche, Villa Farnesina

Construction for the Villa Farnesina was initiated in 1505 for Angostino Chigi, a wealthy and well-connected financier. The interior was decorated between 1515 and 1518 (Gerlini, 2000). On the south side of the villa and within the Loggia di Amore e Psiche are frescoes designed by Raphael Sanzio (1483–1520) and executed by his assistants including Giulio Romano and Giovanni Martini da Udine (1487–1564). The latter individual painted the festoons that contain exquisite images of most of the economically important plants of the region at that time. Caneva (1992) examined the festoons and determined that at least 160 different species of plants and fungi are shown. While leaves, flowers, and fruits are illustrated, only rarely are flowers and fruits of the same species shown together. For the most part, the vegetative material portrayed in the festoons is unrelated to the flowers and fruits. The images provide a snapshot of the various fruits and vegetables that would have been present in the vicinity of Rome in the early 16th century, and these are illustrated in intricate detail. For example, codling moth damage can be seen on some of the apples and mold is present on one of the melons. The images are considered a prototype for realistic still-life paintings of the Renaissance (Janick and Paris, 2006).

**FIGURE 2:**
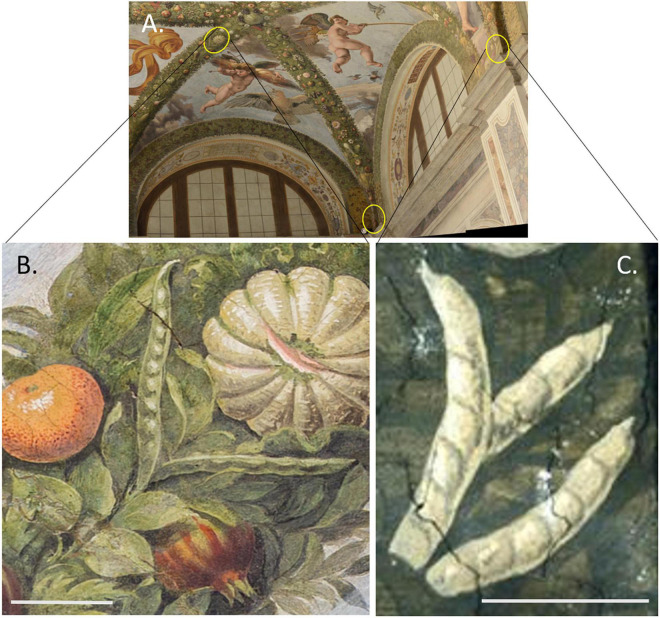
Overview of the Loggia of Cupid and Psyche in the Villa Farnesina and close up of legume pods. **(A)** View to the northwest with the location of putative common bean pods indicated by yellow circles in the festoons. Source: Sgamelotti and Caneva (2017). **(B)** Pods subtending a melon near the fresco depicting Cupid with the aspects of Jupiter. Source: J. Janick. **(C)** Pods located at the Foot of the Three Graces. Size bars = 10 cm. Source: J. Janick.

Caneva (1992) found one or more images of seven species of legumes in the festoons (Supplementary Table 1). Most images were of the fruit, but one (clover) depicts solely flowers. Flowers of *Vicia faba* in association with pods are also present. None of the legume pods appear to have leaves of that species associated with them. Caneva (1992) determined that three depictions were probably that of common beans. The location of these images in the Loggia is shown in Figure 2A.

In the first set, one pair of pods subtends a *Cucumis melo* fruit in the festoons to the right of the panel showing Cupid with the Attributes of Jupiter (Figures 2A,B). The pods are arranged such that the bases are overlapping; one pod is visible to the pedicel, with the base and pedicel of the other just visible beyond the overlapping pod. The bases meet below the melon and the apices are arranged to either side of the melon. Taking as a base measurement of 10 cm for the pomegranate located below and to the right of the pods, we obtained a length of 21 cm for the rightmost pod and 22 cm for the left pod, and widths of 2.0 and 2.2 cm, respectively (Table 3). Pods consist of single locules that are laterally compressed, with slightly embellished margins. The apices and base of one pod are tapered. The apices are centrally located with a spur present with apex central and spur straight to slightly curved. Seed cavities are apparent on the outside of the pod showing up to nine ovules. The length: width ratio is ≥9 for both pods. Pods appear to be green as if fresh material was used as a model.

**TABLE 3:** Legume pods in the festoons of the Loggia di Amore e Psiche in the Villa Farnesina compared to large-seeded legume genera.

Image or plant species	Pod length (cm)	Pod width (cm)	Length:width ratio	Seeds/pod (no.)
**Adjacent to Cupid with the Attributes of Jupiter**				
Left pod	22	2.2	10	9
Right pod	21	2.0	10	5
**Near the Foot of the Three Graces**				
Upper pod	>16	2.7	>6	7
Middle pod	>10	2.6	>4	3
Lower pod	>13	2.7	>5	6
**Plant species**				
*Phaseolus vulgaris*	8–18	1.0–1.5	≥8	4–12
*Albizia spp.*	7–38	0.7–3.6	≥9	3–15
*Canavalia spp.*	7–40	1.5–6.0	≥9	3–15

The second set of three pods is found at the Foot of the Three Graces (Figures 2A,C). The basal region of all of the pods is obscured, so an accurate length cannot be obtained. What is visible ranges from 10 to 16 cm based on scaling to a pomegranate located above the pods. Pod widths range from 2.6 to 2.7 cm and the length: width ratios range from four to six. The pods consist of a single laterally compressed, adaxially curved locule. The apex tapers abruptly into a straight to slightly curved spur. Spurs are located placentally in two pods and centrally in the third. Obliquely oriented seed cavities are apparent on the outside of the pod. There is slight compression between three to seven ovules that are visible. Pods are tan in color suggesting that they were dry at the time the artist viewed them, rather than using fresh material that is green in color.

The third set of pods is located at the base of the northwest corner groin (Figure 2A). The pods wrap around the corner and as a result, are distorted in images available for analysis. Based on a pomegranate located above the pods, pods appear similar in color, size, and shape to those located at the Foot of the Three Graces, but accurate measurements cannot be obtained from images available to us.

**FIGURE 3:**
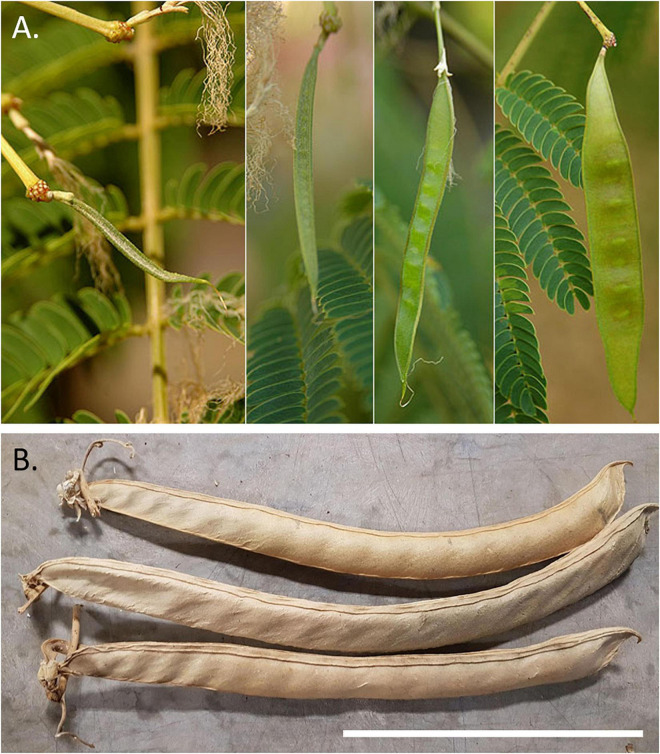
Possible species contributing the large legume pods found in the Loggia of Cupid and Psyche in the Villa Farnesina. **(A)** Silk tree (*Albizia julibrissin*) pods showing different stages of development. Photo: P. Breen, Landscape Plants, Oregon State University. (https://landscapeplants.oregonstate.edu/plants/albizia-julibrissin). **(B)** Dry pods of Sword Bean (*Canavalia gladiata*). Photo: J. R. Myers. Size bar = 10 cm.

A comparison of the characteristics of the three sets of pods reveals that the two are like one another, but quite different from the set subtending the melon. At 21–22 cm long, the pods of the latter near the panel with Cupid with the Attributes of Jupiter extend beyond the upper range in length for *P. vulgaris* (Table 3 and Supplementary Table 2). Some *P. coccineus* pods may reach this length but in addition, *Phaseolus* spp. in general, lack embellished margins and prominent seed cavities. This set has characteristics that more closely resemble tree species in the Mimosidae subfamily with InKey pointing to genus *Albizia*. *Albizia julibrissin* or Silk Tree (Figure 3A) is an Asian species that was distributed globally as an ornamental quite early on; it was brought into American gardens in 1745 (Miller, 1999), and presumably earlier in Europe. It is plausible that this or a related species was growing in the viridarium of the villa and supplied the pods at the time of rendering of the festoons. Chigi’s viridarium was well known to the Vatican in the 16th century for its collection of rare plants (Janick and Caneva, 2005).

The pods at the Foot of the Three Graces as well as those on the northwest corner groin are similar in appearance and superficially resemble those of *Phaseolus* spp. The full length cannot be distinguished but what is visible at 16 cm is trending toward the upper range for *Phaseolus* spp. In addition, the pod width at >2.5 cm is outside the expected range for *P. vulgaris* (Table 3 and Supplementary Table 2). If we assume a pod length to width ratio of nine, then based on pod width, we would predict a total pod length of around 24 cm. Given the pod size and shape and the obliquely oriented seed cavities, the closest candidate according to InKey is the *Canavalia* genus. This genus consists of about 50 species, four of which have been domesticated, and two which have been distributed worldwide. *Canavalia ensiformis* is originally from the subtropical Americas and *C. gladiata* comes from the Asian subtropics (Sauer, 1964). Of the two, *C. gladiata* comes closest in appearance to the Villa Farnesina pods (Figure 3B). This species possesses pods up to 40 cm in length with oblique-elongate ovules. Mature pods are rather fibrous and may or may not be dehiscent. Once dried, the pods are very persistent. One of the authors (Myers) has kept a pod specimen that has remained intact for more than 30 years. The species is widespread from India to Southeast Asia, China, and Japan. While adapted to the subtropics, it has been grown at latitudes of up to about 40°N, where the growing season is long enough (Sauer, 1964). It was described taxonomically in the 18th century from SE Asian specimens, but there is evidence that it was distributed early on to the Americas as well as being grown in Europe. Our hypothesis is that the images in Villa Farnesina are modeled after *C. gladiata* pods that came from plants grown in Villa’s viridarium, or from pods brought back from Asia.

### Herbals and Other Images

**FIGURE 4:**
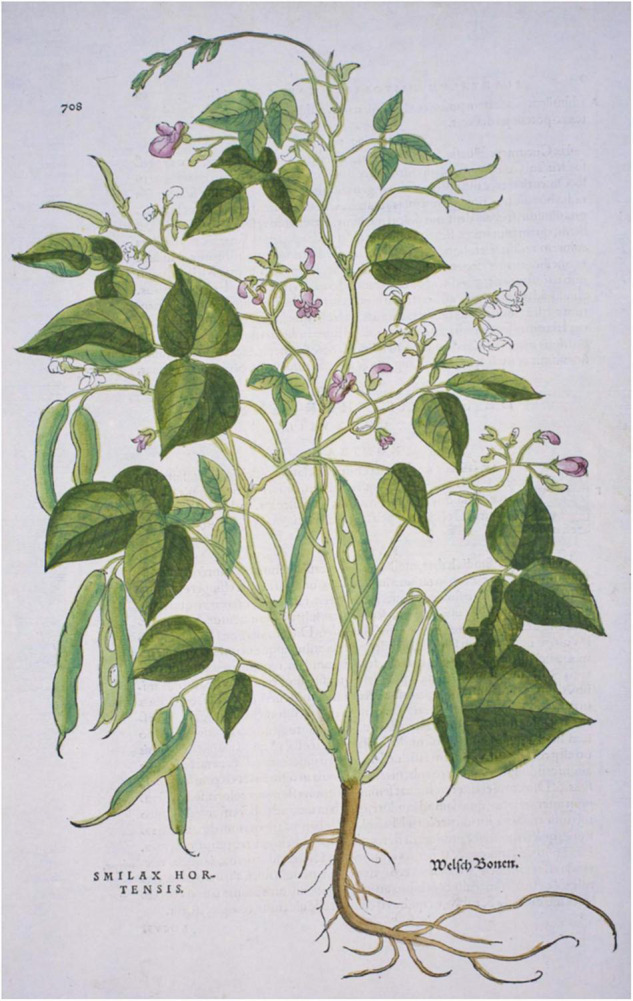
Folio 708 from Leonard Fuchs (1542) *Di Historia Stirpium* showing an image of common bean (*Phaseolus vulgaris*). Source: Public domain; Hunt Institute for Botanical Documentation.

Prior to Leonhart Fuchs’ (1542) publication of an illustration of a common bean plant in *Di Historias Stirpium*, no other images have been found. Otto Brunfels’ herbals depict no New World crops or mention that any crops came from America; however, his *Herbarium* (Brunfels, 1532b) and his *Contrafeyt Kreuterbuch* (Brunfels, 1532a) mention and briefly describe a climbing, kidney-shaped “welsh bean”, where “welsh” was understood to mean “foreign”, and more particularly of French or Italian origin (Brücher, 1989). In the *Kreuterbuch*, he noted that the seeds were different colors and that it was a recent arrival, that Hieronymus Bock had identified as *Smilax hortensis*, but Brunfels was unsure of this interpretation (Johnson, 2008) but ultimately deferred to Bock. In his herbals of 1532 and 1536 Brunfels, 1536), there is a caption for an illustration of this bean, but the plant depicted is a *Silene vulgaris* and does not match the description, so this was likely an error that was propagated across various editions of his work. Hieronymus Bock (1539) also provided some description but no illustrations of the climbing “welsh bean” (Supplementary Table 3). A decade after Brunfels’ herbal was published, the *Di Historias Stirpium* (Fuchs, 1542) had woodcuts of maize, squash, tomato, pepper, and common bean. This first image (Figure 4), of what is clearly a common bean, was also described by Fuchs as “welsh bean”. The plant depicted in Fuchs’ herbal has indeterminate (type IIIA) growth habits (Singh, 1981) and medium-sized leaves and pods. It is clearly a common bean based on the presence of bracteoles at the base of pods and flowers, as well as from the general shape of leaves and pods (Figure 4).

**FIGURE 5:**
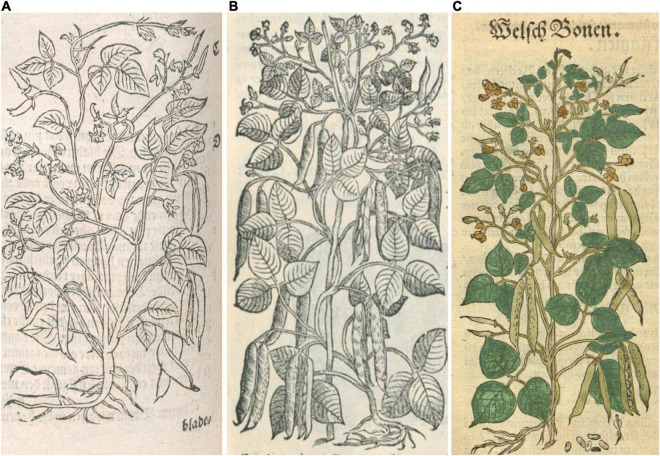
Woodcuts of common beans from 16th-century herbals. **(A)** Dodoens (1563), *Cruÿdeboeck*… Source: Public domain; Wellcome Library, London, United Kingdom (Proquest). **(B)** Bock (1546), *New Kreuterbuch.* Source: Public domain; Biodiversity Heritage Library (Holding Institution: Missouri Botanical Garden, St. Louis, MO, United States)**. (C)** Mattioli (1558), *Medici, commentar secondo aucti*… Source: Public domain; Universitats- und Landesbibliothek Dusseldor DE.

**FIGURE 6:**
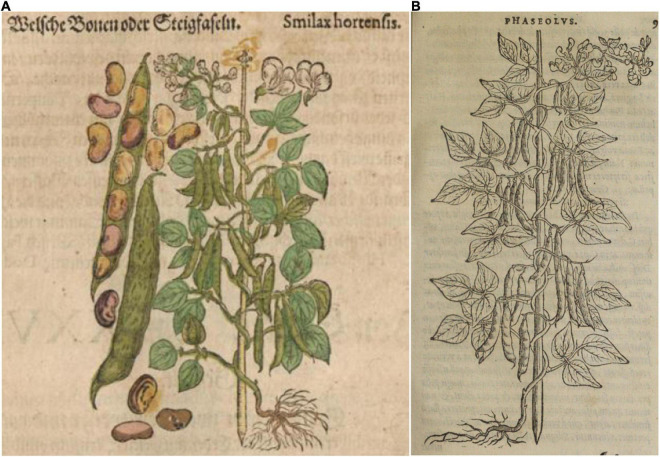
Woodcuts of common beans from 16th century herbals. **(A)** Mattioli (1590) *Kreutterbuch deβ hochgelehrten unnd weitberühmten*… This illustration is a reverse image of panel **(B)**, with the addition of pods, seeds, and flowers. Source: Public domain; Universitats- und Landesbibliothek Dusseldor DE. **(B)** Dodoens (1566), *Frumentorum, leguminum, palustrium et aquatilium herbarum*… Source: Public domain; Wellcome Library, London, United Kingdom (Proquest).

**FIGURE 7:**
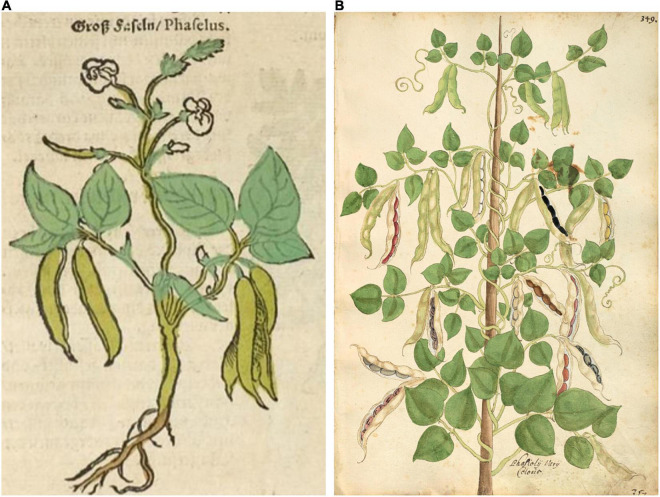
Woodcuts of common bean from 16th century herbals. **(A)** Roesslin (1550). *Kreuterbuch.* Source: Public domain; National Library of the Czech Republic (Google Books). **(B)** Oellinger (1553), *Magnarum Medicinae partium herbariae*… Folio 349, *Phasioli Vary Coloris*. Source: Public domain; Universitätsbibliothek Erlangen-Nürnberg, DE.

**FIGURE 8:**
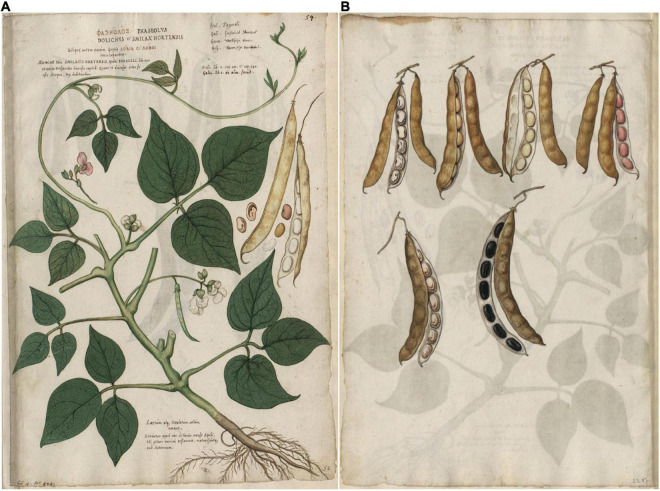
Two plates from *Libri Picturati (1550–1595)* of common beans. **(A)** Plant with a detached pod. **(B)** Pods from different plants showing diversity in seed and pod shape and color. Source: Public domain; Biblioteka Jagiellońska, Kraków, PL.

**FIGURE 9:**
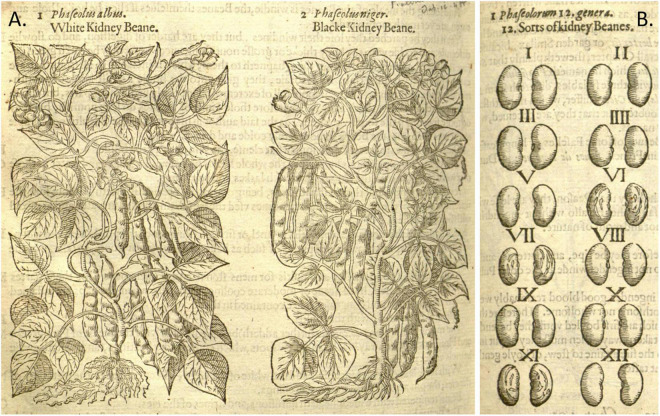
Woodcuts of Phaseolus beans from Gerard’s (1597) *Herbal*. **(A)** Plate showing two common bean plants labeled White and Black Kidney. **(B)** Seeds of “kidney Beanes”. Source: Public domain; Biodiversity Heritage Library (Holding Institution: Missouri Botanical Garden, St. Louis, MO, United States).

**FIGURE 10:**
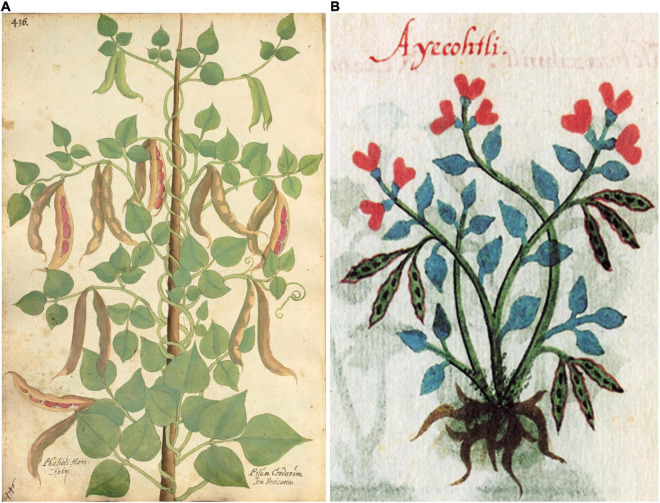
Woodcuts of Phaseolus beans from 16th century herbals and manuscripts. **(A)** Oellinger (1553), *Magnarum Medicinae partium herbariae*…, Folio 436, *Phasioli hort* (*Phaseolus vulgaris*). This illustration shows a red-brown seed and may represent the seed color of the original specimen. Source: Public domain; Universitätsbibliothek Erlangen-Nürnberg, DE. **(B)** *Codex Cruz-Badianus* showing a phytomorph of *Phaseolus coccineus*. Source: Tucker and Janick (2020).

Nine additional illustrations from eight authors representing depictions of common beans in the 16th century were compared to Fuchs’ image (Table 4 and Figures 5–10). The images of German origin included Fuchs (1542), Bock (1546), Roesslin (1550), and Oellinger (1553); from France: Libri Picturati (1550–1595); from Belgium: Dodoens (1563, 1566); from Italy: Mattioli (1558, 1590); from England: Gerard (1597). Half of the illustrations were monochrome woodcuts, but most of the remainder were woodcuts that had been hand-painted. The illustrations were analyzed for eight traits (Supplementary Table 4) diagnostic of the centers of domestication and races of common bean (Singh et al., 1991). Figures 5A–C show woodcut images of varying quality of early illustrations, especially compared to Figure 4. After classification, the illustrations were compared to identify duplicates. We found that Dodoens (1563; Figure 5A) was a reverse image of Fuchs (1542; Figure 4) and Mattioli (1590; Figure 6A) was a reverse image of Dodoens (1566; Figure 6B). Mattioli’s (1590) illustration also differed in having isolated depictions of flowers, pods, and seeds not drawn to the same scale and surrounding the bean plant (Figure 6A). Classifications for duplicate pairs among traits were similar with minor variations, mainly in leaf shape and flower and seed numbers, but the overall classification to the center of domestication and race was the same. Going forward, the duplicate pairs will be considered as one for discussion purposes.

**TABLE 4:** Comparison of early common bean herbal illustrations for traits associated with centers of domestication and races of common bean.

Herbal illustration	Growth habit	Leaf size	Central leaflet shape	Bracteole size and shape	Spur position	Flowers or pods/raceme (no.)	Seeds/pod (no.)	Seed shape	Center of domestication (putative race)
Fuchs, 1542	IIA or IIIA	Medium	Cordate-ovate	Large	Placental	2–4	3–4	Oval	Middle-American (Mesoamerica)
Bock, 1546	IIIA or IV	Small	Cordate	Large	Placental	2–4	6	Oval	Middle-American (Mesoamerica)
Roesslin, 1550	IIIA	Medium-large	Ovate (long)	Small	Placental & central	1–4	3–4	Oval-cylindrical	Andean (Nueva Granada)
Oellinger, 1553	IB (but strongly twining)	Small	Cordate	None present	Placental	2	4–5	Oval-cylindrical	Middle-American (Mesoamerica)
Mattioli, 1558	IB (but twining)	Small	Ovate	Large	Placental	2–5	4–6	Oval-cylindrical	Middle-American (Mesoamerica)
Libri Picturati, 1550–1595 ^1^	IIIB	Small	Cordate	Large, cordate	Placental^1^	2–4	5–7	Oval, cylindrical, kidney, rhombo-hedric	Middle American (Mesoamerica)
Dodoens, 1563	IIIB	Medium	Ovate-hastate	Large	Placental	2–5	3	Oval	Middle-American (Mesoamerica)
Dodoens, 1566	IB (but twining)	Medium	Ovate-haustate-rhombo-hedric	Small	Placental	2–7	4–6	No visible seeds	Andean (Nueva Granada)
Mattioli, 1590	IB (but twining)	Medium	Ovate	Small	Placental	2–8	6	Oval-kidney	Andean (Nueva Granada)
Gerard, 1597 ^2^	IB (long internodes)	Medium	Cordate-ovate	Large	Placental	3–5	5–6	Kidney?^2^	Middle-American (Mesoamerica)

*^1^There are two plates in this manuscript, one showing the plant and a detached pod, the other consisting of six different pods (four in open and unopen pairs, two as single opened pods). Seed and pod types point to a combination of centers of domestication. The pod shown with the plant appears to be from the Andean center of domestication, with a long pod and cylindrical to kidney-shaped seed. The pods of the second folio vary from oval to cylindrical, and one pod with long cylindrical black seeds has a centrally attached spur (others are placental).*

*^2^Gerard’s herbal has images of two “kidney” bean plants that appear to be Phaseolus vulgaris. No seeds are visibly associated with the plants but there is a separate plate showing “Phaseolorum… Sorts of kidney Beanes” that depict seeds with cylindrical to kidney shape.*

Half of the illustrations (Fuchs, 1542; Bock, 1546; Roesslin, 1550; Libri Picturati, 1550–1595; Dodoens, 1563) were clearly indeterminate and most likely Type II, III, or IV (Singh, 1981; Supplementary Table 4 footnote). The other illustrations (Oellinger, 1553; Mattioli, 1558, 1590; Dodoens, 1566; Gerard, 1597) showed plants terminating in inflorescences or pod clusters characteristic of determinacy (Type I) in common beans. However, these were generally shown as weakly to strongly twining, with long internodes, which is not characteristic of modern type I beans that have short internodes and do not twine. When grown under low light levels, determinate beans will etiolate and some (especially older heritage cultivars) will exhibit twining tendencies. In addition, some South American accessions have been observed to be determinate but are type IV climbers (Singh, 1988). Differences in maturity may also have affected the perceptions of growth habits. The Roesslin (1550) image (Figure 7A) appeared rather young, with only about five internodes of an indeterminate plant shown. The Oellinger (1553) image (Figure 7B) shows a very mature plant that has ceased blooming. At this stage in the development, the terminal bud may have senesced and abscised, making it difficult to determine determinacy in older vines. Thus, our classification of type IB for this image is tentative.

We were unable to classify bracteole shape in all illustrations with the exception of Libri Picturati (1550–1595; Figures 8A,B) where the image was exquisitely detailed. However, differences in size were used in combination with other traits to narrow the classification. Where pods were open, the number of seeds per pod could be counted directly, and in intact pods, the number could be inferred based on ovular constrictions. The classification as to seed shape was somewhat problematic because in most cases, only a portion of the seed was visible. We could classify the seed shape as oval or cylindrical but may have missed some kidney-shaped seed because the hilar region of the seed was not visible.

Based on all characteristics (Table 4 and Supplementary Table 4), we found a mix of seven Middle American and three Andean types depicted in the illustrations. The earliest illustrations (Fuchs, 1542; Bock, 1546) appear to be race Mesoamerica, as do the Oellinger (1553) and Mattioli (1558) images (Table 4). The Roesslin (1550) illustration (Figure 7A) was the first to show race Nueva Granada characteristics, as does the image from Dodoens (1566; Figure 6B). Mattioli (1590; Figure 6A) was also classified as Andean but is a copy of the Dodoens (1566) illustration. Some illustrations appeared to represent more than a single race/center of domestication. For example, both Libri Picturati (1550–1595; Figures 8A,B) and Gerard (1597; Figures 9A,B) showed plants of Middle-American extraction, but seeds that varied or that were clearly Andean in shape.

There is some indication in both the writings of these authors as well as from the flowers and seeds shown in these illustrations that characteristics of *P. coccineus* may have been combined with *P. vulgaris*. Fuchs (1542, 1543) noted red flowers and “red or skin-colored seed with black spots” that is characteristic of *P. coccineus* (Zeven, 1997). Bock (1546) has an illustration with a mix of red and white flowers on the same plant in some copies (Supplementary Table 3). Roesslin (1550) provides a similar description of the flowers and seeds. Mattioli (1558; Figure 5C) has a plant hand-colored with red flowers. In Oellinger’s (1553) herbal, Folio 349 (*Phasiolis Vary Coloris*) which illustrates various seed colors (Figure 7B), some show colors and patterning clearly associated with *P. coccineus*. This image is duplicated as Folio 436 with uniform red-brown seeds (Figure 10A). Among the seeds added to the Mattioli (1590) illustration, there are several that resemble *P. coccineus* in color and patterning (Figure 6A). What is probably the first unambiguous depiction of *P. coccineus* is found in the Codex Cruz-Badianus, 1552 (Figure 10B), produced in the Colegio de Santa Cruz in Tlatelolco, New Spain (Tucker and Janick, 2020). The Codex was written by Martin de la Cruz and translated into Latin by Juan Badiano, both indigenous Nahuan staff members. The Latin manuscript was sent to Spain where it was housed in the royal library until the 17th century. The images of *P. coccineus* in the Codex are stylized phytomorphs, but clearly shows the trifoliolate leaves, red flowers, legume pods, and tuberous roots characteristic of this species. In this image (Figure 2.60, p. 100 in the *Flora of the Codex Cruz-Badianus*), the plant is named *Ayecohitli* but a second image in the Codex (Figure 146, p. 218) shows *P. coccineus* with the name *Cimatl* (Tucker and Janick, 2020).

## Discussion

### *Grandes Heures d’Anne de Bretagne* and Villa Farnesina

Folio 194 of the *Grandes Heures d’Anne de Bretagne* has had a rather volatile history. de Jussieu (1722) first described the image as *Phaseolus flore luteo* but Camus (1894) later reclassified it as *P. vulgaris* (Lioi and Piergiovanni, 2013). Apparently, Camus regarded his finding as inconclusive (Bonnet, 1897), and Bonnet suggested that the plant represented in the image may have been a *Vigna* spp. Bilimoff (2001) noted the remark of Camus concerning *P. vulgaris*, but then mentioned that the image also resembles *V. unguiculata* (“mongette”). While *V. unguiculata* may also be considered a candidate, it belongs to the subgenus Vigna and accessions of this subgenus have white to pink flower colors. Yellow nectar guides may be found on the banner petal, but solid yellow flowers are not found within this subgenus. The illustration may have been painted without the benefit of the fresh plant material, or another yellow-flowered legume species may have been used as a model to flesh out the memory of a particular grain legume for which a living material was unavailable. Assuming that Folio 194 was based on a real plant, and is not a chimeric mix of various legume species, the best candidate is a subgenus Ceratotropis *Vigna* species.

Maize and squash are indisputably present in the Loggia di Amore e Psiche, providing evidence that New World crops had been introduced into Italy by the second decade of the 16th century (Janick and Caneva, 2005; Janick and Paris, 2006). In studying the maize images, Janick and Caneva (2005) found that while they were undoubtedly depictions of that species as shown by the shape and the presence of silks, there were botanical inaccuracies in the arrangement of kernels on the ear, and may have been painted from memory, or from an inaccurate preliminary study. Janick and Paris (2006) reviewed the cucurbit images and concluded that three New World squash species (*Cucurbita pepo*, *C. moschata*, and *C. maxima*) were illustrated. Although we agree that *Cucurbita* species are present in the Loggia, we question whether *C. maxima* was present as it was unlikely to have been found in the Caribbean at the beginning of the Columbian exchange (Formiga and Myers, 2019). Both the maize and squash images are displayed prominently in the festoons. By contrast, the legume pod depictions are rather obscure, found only in the northwest portion of the Loggia, and lack key diagnostic details. Their scarcity in the Loggia compared to the other New World species suggests that they were less common in the gardens and markets of the surrounding region. It would be interesting to know the order in which the festoons were painted and whether the time of year coincided with plant growth and reproduction. Based on the pod morphological details we were able to discern, we conclude that the common bean is not represented in the Loggia. Rather, there appear to be at least two different species that had originally been identified as common beans (Caneva, 1992). Based on pod diagnostic features, we believe that one set comes from a subfamily of the Mimosidae species such as *Albizia julibrissin* and another is a depiction of *C. gladiata*. Both are Old World species so our ultimate conclusion is that no New World grain legumes are represented in the Loggia. If neither of these illustrations is of common bean, then what are the first records of introduction into Europe?

### Earliest Records of Common Bean in the Old World

The earliest written record is reported as 1532, where Canon Piero Valeriano was given beans by Pope Clement VII for the purpose of introduction into Italy (Toussaint-Samat, 1987; Papa et al., 2006; Lioi and Piergiovanni, 2013). This information was recorded in a detailed and lengthy poem (Perale, 2001) that Valeriano dedicated to Duke Alessandro de Medici. While written in 1532, the manuscript was not published until 1550 (Birri and Coco, 2000). The Pope was reported to have received the seeds from the Spanish Emperor Charles V. They were described as large-seeded, typical of Andean types, and are reputed to be the source of beans that spread through northern Italy. While detailed descriptions are not available, there may have been an introduction of more than one type because Valeriano’s poem describes beans of many different colors (Perale, 2001). The beans were said to be vining and with climbing ability. In the region today, the Borlotti type “Lamon” is reputed to be a descendent of this introduction (Albala, 2017). Borlotti types are members of the race Chile, which would have originally come from the Pacific side of the South American Andes. Francisco Pizarro explored Peru beginning in 1528, with introductions from this region probably happening soon after (Berglund-Brücher and Brücher, 1976). Thus, some of the beans that Valeriano planted might have been from Peruvian introductions. Embedded in the poem is the mention of giving beans to Catherine di Medici (Perale, 2001), who in 1533 and at the urging of Valeriano, carried a bag of common bean seeds to Marseilles as part of her dowry upon her marriage to Prince Henry, Duke of Orléans and Dauphin of France (Toussaint-Samat, 1987). Along with the first herbals to mention New World beans starting in 1532, these writings suggest that common bean was well established in Europe by the early 1530s.

The gap between when Columbus first mentioned common bean and when it entered written records in the 1530s leads to an additional set of questions: was common bean introduced into Europe at a much later date and diffused quickly, or was it introduced early along with maize and squash, but diffused slowly from the Iberian Peninsula into southern and subsequently northern Europe? The records of maize and squash would suggest that the diffusion happened rapidly. If this was the case with common bean, and it was introduced at least informally after the return of Columbus from his expeditions to the Caribbean, then why is there such a gap in time until scholars recognized and wrote about it as a species distinct from Old World crops? Some scholars believe that the familiarity with cowpea led to common bean being accepted as just another kind of “cowpea”, without recognizing its distinctness or origins (Piergiovanni and Lioi, 2010). The introduction of New World crops also coincided with a revolution in European herbals. Before Brunfels, Bock, and Fuchs published their botanical works in the 1530s and 40s, herbalists were constrained in thought by the idea that all knowledge about plants was derived from the ancient Greeks and Romans. Another factor may have been that the Spanish explorers of the New World were focused on commerce and not on academic pursuits. Few if any scholars accompanied early expeditions and this may also be the reason why we did not find bean illustrations from 16th century Spanish sources other than the Codex Cruz-Badianus.

### Beans in the Caribbean in 1492

What kinds of beans were likely encountered as the Spanish explored the Caribbean and adjacent mainlands? Maize was introduced to Europe with the return of the first expedition as documented in the letter of Peter Martyr to Queen Isabella (Sauer, 1966), but there is no mention of beans. Some scholars have suggested that beans were introduced after Columbus’ second expedition but others have noted that *Phaseolus* spp. beans were probably not introduced into Europe until after 1506 (see for example Angioi et al., 2010; citing Sauer, 1966). Bean and squash seeds were almost certainly introduced informally at the same time as maize. Bean seeds, with the variability in colors and patterns, would have been particularly interesting to New World explorers. Complicating the picture, at least two, and possibly three species of bean (*P. vulgaris*, *P. coccineus*, and *P. lunatus*) were growing concurrently in the Caribbean. Encounters by explorers with *P. coccineus* would have been less likely because this species is adapted to cooler tropical production zones, and would most likely have been only grown at high altitudes in Latin American and interior uplands on the Caribbean islands. In addition to common beans, lima beans were prevalent in the Caribbean because of their adaptation to the hot, humid lowland tropics. Both species would have been taken back to Europe, but common bean would have been better able to transition to a new temperate environment at a higher latitude (Cortés et al., 2013; Cortés and Blair, 2018; López-Hernández and Cortés, 2019), and would have had greater success in subsequent propagation in Europe. There were probably repeated introductions into southern Europe as the Portuguese and Spanish expanded their activities in the Caribbean, and began exploration of the mainland of Central and South America.

Day length sensitivity was likely a factor in successful introductions of all domesticated bean species. Many bean accessions of tropical or subtropical origin require short days to initiate flowering. When these accessions are brought to higher latitudes, they do not begin to flower until late in the growing season and may fail to mature seed. For common beans, day length sensitivity varies by accession, but in general, accessions in races Mesoamerica, Durango, Nueva Granada, and Chile have a greater chance of being day length insensitive, and thus would have been preadapted to a successful introduction into Europe (Singh et al., 1991). Day length sensitivity may have been an issue with lima bean, but this species also requires a long growing season and warmer temperatures, which may have created additional barriers to introduction (Bitocchi et al., 2017).

Europe now represents a secondary center of diversity for common bean, with a bias toward Andean types found in most regions (Gepts and Bliss, 1988; Lioi, 1989a,b; Limongelli et al., 1996; Escribano et al., 1998; Santalla et al., 2002; Rodiño et al., 2003; Lioi et al., 2005; Angioi et al., 2010). In Spain and Portugal where the first introductions of common beans are thought to have happened, Andean types overwhelmingly predominate. In Europe overall, about one-third of accessions are Middle American (S phaseolin) while the Andean two-thirds are split almost equally between T and C phaseolin (characteristic of races Nueva Granada and Chile, respectively) (Angioi et al., 2010). There is a gradient from west to east across Europe with relatively more Middle American types found in the east, but Andean types still predominate. Most prior works that have investigated the introduction of common beans into Europe state that the beans first brought from the Caribbean were small-seeded Middle American types with Andean types arriving only after the 1520s with the initiation of expeditions to the Pacific side of the Andes (Berglund-Brücher and Brücher, 1976). We question this conjecture because landraces from both centers of domestication were very likely present in the Caribbean at the time of Columbus’ expeditions.

By 1492, race Mesoamerica beans had long been disseminated from Latin America into northern South America, where they may have diffused along the Greater and Lesser Antilles and eventually into Hispaniola and Cuba. Alternatively, they may have been introduced directly by trade routes from the Yucatan peninsula or across the Florida straits to the Caribbean islands. Landraces from the Andean center of domestication were likely introduced to the Caribbean islands from South America in a similar manner to Middle American beans. Arawaks were the main population of Native Americans that the Columbus expeditions encountered in Hispaniola and Cuba. This group migrated from the eastern slopes of the Andes in South America and through the Lesser and Greater Antilles to populate the Caribbean islands and arrive in Cuba about 2,940 BP (Castiñeiras et al., 1991; Rouse, 1992). In historical times, the common bean types associated with this group have been of Andean origin represented by the large-seeded, often red, pink with red flecking, and red-mottle types (Beaver and Molina, 1997; Duran et al., 2005). These are race Nueva Granada, with large kidney- or cylindrical-shaped seeds. This body of knowledge indicates that Andean types would have been present at the time of the Columbus expeditions.

The Arawaks displaced other Native American groups that had previously settled the Caribbean islands. One such group is the Guanahatabey who were found only in the western portion of Cuba by 1492. The Guanahatabey people show the greatest genetic affinity to Native American groups in Latin America, and ancient DNA studies have shown that they originally migrated from that region (Fernandes et al., 2021). The bean preferences existing today on the island of Cuba show a clinal gradient where populations in the western third have traditionally used small-seeded black (race Mesoamerica) beans while populations in the central and eastern regions prefer large-seeded Nueva Granada types (Castiñeiras et al., 1991). The Guanahatabey observed by the early Spanish were not thought to practice agriculture (Sauer, 1966), but archeological evidence shows that this group did consume and presumably cultivate beans, but perhaps not on the scale of agriculture that the Arawaks practiced (de Armas et al., 2015). We cannot know for sure whether patterns of human population distribution are related to bean preferences. It may have been that the Guanahatabey had brought Middle American beans with them to the island, and at one time practiced more extensive agriculture but were forced to hunter/fisher-gatherer subsistence as they were driven into more barren and less productive western Cuba. A similar distribution of beans was historically present on Hispaniola, where small-seeded Middle American types were confined to the west and large-seeded Andean types were cultivated in the highlands and eastern portions of the island (Duran et al., 2005). Unlike the case of Cuba, a distinctly separate human population was not observed, but ancient DNA studies do show that some populations on Hispaniola, particularly to the west, had a mixture of archaic and ceramic people DNA (Fernandes et al., 2021).

When Columbus visited the present-day shores of Honduras in 1502 and reported seeing beans, the types he would have encountered would have almost certainly been race Mesoamerica small reds and whites (and possibly blacks, although only red and white seed were described by Ferdinand Columbus; Sauer, 1966). Whether Columbus or any of his men brought back beans from that expedition is debatable as he was shipwrecked and returned to Europe destitute and in disgrace. Many expeditions by others followed with opportunities to acquire beans and introduce them to the Old World.

**FIGURE 11:**
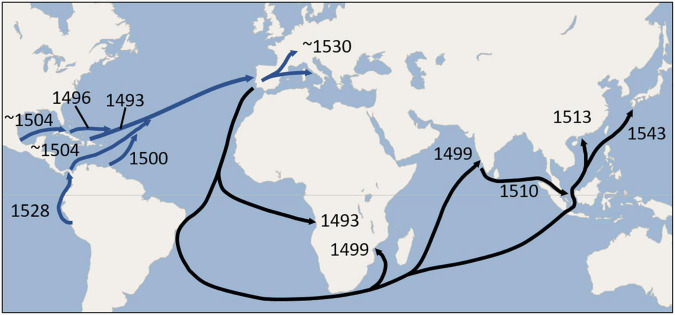
Possible routes and earliest dates for the movement of *Phaseolus* beans from the New World to Europe (blue arrows), and earliest possible dates for dissemination to Africa, India, and Asia (black arrows) during the 16th century. The routes depicted are mainly those of the Spanish and Portuguese who were the earliest to establish the trade routes. Exploration and trading along the African Atlantic coast predated the Columbian Exchange, but the earliest possible date for the introduction of beans from Europe would have been 1493. Introductions to Africa and Asia may have been carried directly from the New World as well as from Europe. Return voyages by ships engaged in the slave trade from the New World directly to Africa may have been particularly instrumental in introducing common bean to that continent.

Europe’s status as a secondary center of diversity for common beans (Santalla et al., 2002; Angioi et al., 2010; Lioi and Piergiovanni, 2013; Bitocchi et al., 2017) has come about from repeated introductions of this species over time from the different gene pools of the New World. Within Europe, the intermixing among different races and especially different centers of domestication has greatly exceeded any intercrossing observed in the Americas. One product of the intermingling of gene pools is snap bean, which Native Americans seemed not to have in abundance, but flourished in Europe (Blair et al., 2010; Wallace et al., 2018). These along with new varieties of dry beans developed in Europe were exported back to North America, to the African continent (Asfaw et al., 2009), India, Southeast Asia, China (Wu et al., 2020), and Japan. Figure 11 provides a global map that shows putative earliest dates for the movement of common bean to Europe and their possible distribution from Europe to the rest of the Old World.

## Conclusion

We find that the first hard evidence of the introduction of beans into Europe comes from 1532 and thereafter and that the visual evidence from prior to 1530 that has entered the literature does not actually represent common beans. The gap in time between when common beans were first observed in the Americas and when they entered the literature in Europe is similar to when maize and squash were observed, and when scholarly works were published on these crops. There were likely repeated introductions, some successful, others not. Diffusion from the Iberian Peninsula to the rest of Europe likely occurred rapidly but informally. The predominance of Andean types in Europe raises additional questions. Early explorers would have encountered types from both centers of domestication and would have brought both types back to Europe. Our examination of early herbal images suggests that both Middle America and Andean types were illustrated, and thus present in Europe simultaneously. The contemporary dominance of Andean types might be a function of adaptation and/or preference. Perhaps Andean introductions had a higher percentage of success in European agriculture. Alternatively, Europeans may have preferred the larger seed size as well as certain cooking and organoleptic properties. In addition, the written record coincides with the penetration of Spanish explorers into Peru where they would have encountered race Chile Andean types.

There are certain incongruities among the herbal images that suggest either an observational bias or the plants of that time were significantly different from those of today. Generally, the leaves and bracteoles depicted are race Mesoamerica, with a lack of leaves and bracteoles typical of race Nueva Granada even in specimens that have other Andean traits. The depiction of determinate plants with long internodes and twining ability is another incongruity. It implies that this trait may have been more common among early introductions compared to today. Several of the drawings, particularly from the latter half of the 16th-century, show kidney-shaped seeds more common among Nueva Granada types, while the plants have Mesoamerican traits. Flower and seed color traits characteristic of *P. coccineus* seem to be represented more often than would be expected based on their productivity relative to *P. vulgaris*. *P. coccineus* is reliant on pollinators or hand tripping to set seed, whereas *P. vulgaris* is self-pollinated. The red flowers and large, colored seeds of *P. coccineus* would certainly draw attention and could be the reason that they were depicted so often.

We have found that iconography can add an additional dimension to the study of crop origins, but much uncertainty remains. One of the best ways forward to understanding the migration of common beans is through the use of molecular markers for genetic diversity studies. A number of such studies have been performed but these have mainly been on disparate populations with marker types that are not easily comparable among studies. For example, we were unable to find manuscripts that allowed us to relate the genetic similarity of accessions from the Caribbean with those found in Europe. Such a study would allow testing the hypothesis that some Andean types grown in Europe originated from the Caribbean rather than South America. One approach would be to resequence accessions from the major centers of interest to understand this and the related questions. We offer the following hypotheses that are testable by examining landrace populations from the various geographical regions: (1) small-seeded beans found on the islands of Cuba and Hispaniola will be most closely related to small-seeded accessions from Mesoamerica and not to those from northern South America and Brazil. (2) Large seeded Nueva Granada landraces from the Caribbean will be most closely related to landraces from Spain and Portugal, with a gradient of increasing genetic distance as one moves east and north across Europe. (3) Landraces of Italy will show the greatest affinity to those from the western slopes of the Andes and not the Caribbean. (4) Beans may have been introduced into Africa directly from the New World through the slave trade as well as indirectly from Europe. A caveat is that repeated introductions may obscure the relationship between regions, but utilization of the oldest landraces may be able to overcome this obstacle.

## Data Availability Statement

The original contributions presented in the study are included in the article/Supplementary Material, further inquiries can be directed to the corresponding author.

## Author Contributions

JM and JJ conceived of the manuscript. JM conducted the analysis of images and wrote the first draft of the manuscript. JM, AF, and JJ researched the topic, obtained historical documents, contributed to revisions of the manuscript, and approved the submitted version.

## Conflict of Interest

The authors declare that the research was conducted in the absence of any commercial or financial relationships that could be construed as a potential conflict of interest.

## Publisher’s Note

All claims expressed in this article are solely those of the authors and do not necessarily represent those of their affiliated organizations, or those of the publisher, the editors and the reviewers. Any product that may be evaluated in this article, or claim that may be made by its manufacturer, is not guaranteed or endorsed by the publisher.
