# Supplementary material for: Iconography of Beans and Related Legumes Following the Columbian Exchange
Source: Front Plant Sci. 2022 Mar 14;13:851029. doi: 10.3389/fpls.2022.851029 (PMC8964180; doi:10.3389/fpls.2022.851029)
Supplement: Supplementary file 1 [file Data_Sheet_1.PDF]

Supplementary Material

1 Supplemental Tables

Supplementary Table 1. Caneva's (1992) classification of Fabaceae in Udine's festoons in the Villa Farnesina.

Common name	Scientific name
Carob	*Ceratonia siliqua* L.
Common bean	*Phaseolus vulgaris* L.
Broad bean	*Vicia faba* L.
Garden pea	*Pisum sativum* L. Ssp. *Sativum*
Clover	*Trifolium* cfr. *Medium* L.
Cassia	*Cassia fistula* L.

Supplementary Table 2. Comparison of pod characteristics among four genera of Fabaceae. Note that the ranges for traits include wild as well as cultivated species.

	**Genera** (Subfamily)			
**Characteristic**	***Canavalia*** (Faboideae)	***Leucaena*** (Mimosoideae)	***Albizia*** (Mimosoideae)	***Phaseolus*** (Faboideae)
Fruit length (cm)	7–40	9–38	5-42	1–18
Fruit width (cm)	1.5–6.0	0.9–3.6	0.5-7.0	0.2–2.0
Fruit thickness (cm)	0.7–3.0	0.2–0.3	0.1-1.0	0.2–1.1
Fruit length: width	Length 2-9x > wide; or ≥ 9 x	Length 2-9x > wide; or ≥ 9 x	Length 2-9x > wide; or ≥ 9 x	Length 2-9x > wide; or ≥ 9 x
Fruit beak position & orientation	Placental, declined; or straight	Medial, straight	Without beak to medial, straight	Placental, declined; or hooked
Fruit apex	Tapered or short tapered	Rounded, tapered or short tapered	Long acuminate to short tapered	Long tapered, tapered or short tapered
Fruit texture	Coriaceous; or ligneous	Chartaceous; or coriaceous; or leathery	Coriaceous to chartaceous, or ligneous	Chartaceous; or coriaceous
Seed chambers (ext. appearance)	Invisible (but may show constrictions)	Visible or invisible	Visible	Invisible (but may show constrictions)
Seed no.	3–15	6–26	8-26	4–20
Seed orientation (seed ln.: fruit ln.)	Oblique or transverse	Oblique or transverse	Parallel	Parallel or transverse
Seed length (mm)	7–48	5–11.8	3-22	1.5–33
Seed width (mm)	5–25	2.7–10.6	2-15	1.5–20
Seed thickness (mm)	2–21	1–2	1-9	1–12
Native distribution Old & New World	New World; or Old World	New World	New or Old World, pan subtropical	New World
Country distribution, (native)	SW Asia, China, Japan, Africa, Madagascar, India, Indochina, Indonesia & Philippines, Australia, New Zealand, Fiji, Hawaii	Mexico to S. US, Caribbean, Central & South |America	Southwest Asia; or China; or Korea; or Africa; or Madagascar; or India; or Indochina; or Indonesia and the Philippines; or Australia	North, Central and South America

Supplementary Table 3. A selection of herbals of the 16th century examined for mention and/or illustrations of *Phaseolus* species.

Author	Document	Date	Comments	Source
Otto Brunfels	*Novi herbarii tomus II*	1531	Latin. No mention of beans or any New World crops found.	Universitätsbibliothek Erlangen-Nürnberg; urn:nbn:de:bvb:29-bv009343581-4
Otto Brunfels	*Herbarum vivae eicones…*	1532	Latin. Mentions "welsh bean" but provides no origin or illustration.	Universitätsbibliothek Erlangen-Nürnberg; urn:nbn:de:bvb:29-bv009343578-7
Hieronymus Bock (Tragus)	*New Kreütter Buch: von Underscheydt, Würckung und Namen der Kreütter…*	1539	German. No illustrations; discusses "Welsch Bonen" & Cowpea ("Phaseoln"). Other potential New World crops include "Welsch Korn" & *Cucurbita* spp.	Münchener Digitalisierungs Zentruim Digitale Bibliothek, Regensburg, Staatliche Bibliothek; urn:nbn:de:bvb:12-bsb11069345-9
Hieronymus Bock (Tragus)	*Kreüter Buch, darin Underscheid, Würckung und Namen der Kreüter …*	1546	German. Has illustrations - some woodcuts are from Brunfels & Fuchs, others including bean image are original. Bean has mix of red & white flowers & colored & white seeds. 1595 ed. shows mix of purple & white flowers.	Biodiversity Heritage Library; Holding Institution: Missouri Botanical Garden, Peter H. Raven Library; DOI: https://doi.org/10.5962/bhl.title.8043
Leonhart Fuchs	*De historia stirpium…*	1542	Latin. 'Welsche Bonen'. "…flowers are white or red, & that the seeds are red or skin-colored with black spots, liver- colored with or without spots, snow-white, white-grayish or yellow." (Zeven, 1997)	Hunt Institute for Botanical Documentation, Carnegie Mellon University https://www.huntbotanical.org/library/show.php?10
Leonhart Fuchs	*New Kreüterbůch…*	1543	German translation. Welsch Bonen & *Smilax hortensis*. Identical to 1542 illustration but not colored.	Universitätsbibliothek Erlangen-Nürnberg; urn:nbn:de:bvb:29-bv008594739-8
Leonhart Fuchs	*Medici, Primi De Stirpivm Historia Commentariorvm…*	1545	Latin. *Smilax hortensis*. Illustration identical to prior editions. Hand colored but faded.	Universitätsbibliothek Erlangen-Nürnberg; urn:nbn:de:bvb:29-bv009196739-5
Eucharius Roesslin	*Kreuterbuch Künstliche Conterfeytunge der Bäume…*	1550 (1569)	German. Published in 1550; 1569 ed. reviewed. "…color of the flower as white, palish yellow or red, & the seeds as red, skin-colored with black spots, leather-colored, white, grey, or golden yellow." (Zeven, 1997).	Originally digitized by the National Library of the Czech Republic; available from Google Books
Libri Picturati	*Des fleurs et fruits [A 23]*	1550-1595	Collection of drawings & paintings by several artists (probably including Jacques van den Corenhuyse & Pieter van der Borcht) with notes by Caroli Clusii. Bean section said to have been created around 1560. Images of bean plant, pods & seeds. Many other legumes & may be 1st herbal showing root nodules of some legumes. See also de Koning et al., 2008.	Biblioteka Jagiellońska, titled as Libri picturati A 23.
Georg Oellinger	*Magnarum Medicinae partium herbariae et zoographiae imagines*	1553	Latin. Two plates of same image, one with red-brown seeds, the other with range of seed colors. Some seed color/patterns resemble *P. coccineus*.	Universitätsbibliothek Erlangen-Nürnberg; UER MS 2362, urn:nbn:de:bvb:29-bv040687699-6
Martin de la Cruz & Juan Badiano	*Codex Cruz-Badianus* (originally *Libellus de Medicinalibus Indorum Herbi*)	1552	Produced in Mexico; Originally written in Nahuatl then translated to Latin. Manuscript sent to the Spain Royal Library. Bean images is *P coccineus*.	Tucker & Janick (2020).
Rembert Dodoens	*Cruÿdeboeck. In den welcken die geheele historie…*	1563	German Described as 'Roomsche Boonkens' (Dutch), 'Welsche Boonen' (German) & 'Phaseolis' (French); "…seed kidney-shaped & colored red, yellow, white, black, or variegated." (Zeven, 1997).	Wellcome Library, London. Bibliographic name/number: 401136.
Rembert Dodoens	*Frumentorum, leguminum, palustrium et aquatilium herbarum …*	1566	Latin. Used Fuchs' woodcuts along with new illustrations.	Wellcome Library, London. Bibliographic name/number: 88525.
Petri Andreae Mattioli	*Medici, commentarii secundo aucti, in libros sex…*	1558	Latin. Described as *Simlax hortensis*.	Universitats- und Landesbibliothek Dusseldor; urn:nbn:de:hbz:061:2-171031-p0001-2
Petri Andreae Mattioli	*Kreutterbuch Desz Hochgelehrten vnnd weitberühmten …*	1586	German. Welsche Bonen & *Smilax hortensis*. Reverse image of Dodens 1566 with addition of pods & flowers. Colored.	Universitätsbibliothek Erlangen-Nürnberg; urn:nbn:de:bvb:29-bv009000910-8
Petri Andreae Mattioli	*Kreutterbuch deß hochgelehrten unnd weitberühmten…*	1590	German. Identical to Mattioli 1586 but better colors to illustrations.	Universitats- und Landesbibliothek Dusseldor; urn:nbn:de:hbz:051:2-126828-p0268-5
Caroli Clusii	*Atrebatis Rariorum aliquot stirpium :per Pannoniam, Austriam, & vicinas quasdam provincias observatarum historia... .*	1583	Latin. Several images of grain legumes, some of which appear to be New World, others are Old World. This herbal may have the first image of *Phaseolus lunatus*, & *P. coccineus* may also be depicted, but no *P. vulgaris*.	Biodiversity Heritage Library; Holding Institution: Missouri Botanical Garden, https://doi.org/10.5962/bhl.title.845
Durante, Castore	*Herbario novo*	1585 (1602)	Italian. Originally published in 1585; 1602 edition reviewed. Image of "Faggiuoli" is small & lacks diagnostic detail.	Universitätsbibliothek Erlangen-Nürnberg; urn:nbn:de:bvb:29-bv008962251-0
John Gerarde	*The Herball, or, Generall historie of plantes*	1597	English. White & black kidney bean are *P. vulgaris*, others are Old World spp. or *P. lunatus*. Separate images of "kidney Beane" seeds.	Biodiversity Heritage Library; Holding Institution: Missouri Botanical Garden, https://doi.org/10.5962/bhl.title.51606

Supplementary Table 4. Selected races of common bean characterized for eight morphological traits, and potentially encountered in the Caribbean and adjacent shores of the Americas by early European explorers. Race Chile would not have been encountered until the Spanish began exploring the Pacific side of South America, but is included here because it is one of the dominant races found in Southern Europe. Table modified from Singh et al., 1991.

	**Race**			
**Trait**	**Mesoamerica**	**Durango**	**Nueva Granada**	**Chile**
**Growth habit**[1]	III (ancestral), also type I and II	III	I, II & III	III
**Leaf size**	Small, medium or large	Small-medium	Large	Small-medium
**Central leaflet shape**	Ovate, cordate or hastate	Ovate or cordate	Hastate, ovate or rhombohedric	Hastate, ovate or rhombohedric
**Bracteole size & shape**	Large cordate	Small ovate (pointed tip)	Small lanceolate or triangular	Small triangular
**Spur position**	Placental (dorsal suture)	Placental	Central	Central
**No. flowers or pods /inflorescence**	Multinoded	Multinoded	Single-noded	Single-noded
**No. seeds/pod**	6-8	4-5	4-6	3-5
**Seed shape**	Oval, cylindrical, kidney	Rhombohedric	Kidney, cylindrical	Oval, round
**Phaseolin type**[2]	S	S	T	C
**Center of domestication**	Middle-American	Middle-American	Andean	Andean
**Distribution in 1492**	Humid lowlands of Latin America & Caribbean	Semiarid highlands of Mexico	Low to intermediate altitudes of Andes, northern South America & Caribbean	Central - Southern Andes

[1]CIAT plant architecture classification (Singh, 1981). I = determinate bush, II = indeterminate upright short vine with weak climbing ability, III = indeterminate sprawling vine with weak climbing ability, IV = indeterminate vine with strong climbing ability. [2]Major seed storage protein in beans. S phaseolin is characteristic of beans from the Middle American center of domestication, while T and C are found in the Andean center of domestication.
